# Internal Grammar and Children's Grammatical Creativity against Poor Inputs

**DOI:** 10.3389/fpsyg.2017.02074

**Published:** 2017-12-01

**Authors:** Adriana Belletti

**Affiliations:** ^1^Département de Linguistique, Université de Genève, Geneva, Switzerland; ^2^DISPOC, Università di Siena, Siena, Italy

**Keywords:** grammatical creativity, grammatical complexity, featural relativized minimality, a-Topic, passive

## Abstract

This article is about the unexpected linguistic behavior that young children sometimes display by producing structures that are only marginally present in the adult language in a constrained way, and that adults do not adopt in the same experimental conditions. It is argued here that children's capacity to overextend the use of given syntactic structures thereby resulting in a grammatical creative behavior is the sign of an internal grammatical pressure which manifests itself given appropriate discourse conditions and factors of grammatical complexity and which does not necessarily require a rich input to be put into work. This poverty of the stimulus type situation is illustrated here through the overextended use of *a*-Topics and reflexive-causative passives by young Italian speaking children when answering eliciting questions concerning the direct object of the clause.

## Introduction

Young children sometimes display an unexpected linguistic behavior: they produce structures that are at most only marginally present in the adult language. This holds both in the sense that the relevant constructions are rarely present in the language and in the sense that their occurrence is severely constrained, as descriptive work clearly indicates. Furthermore, children may react differently from adults to the very same experimental conditions, producing structures that are not “simpler” in any intuitive sense of the term. This type of children's linguistic behavior, which is in fact quite widespread in work on development, indicates that some internal pressure, partly due to factors of computational complexity as we will argue, leads children to be grammatically creative.

The following article is about two case studies with precisely these characteristics, based on experimental results on the acquisition of Italian, recently presented and discussed in detail in Belletti and Manetti (2017). The experiment aimed at studying the acquisition of two different empirical domains in Italian: Romance-type topicalization/Clitic Left Dislocation (ClLD) and types of passive. The first domain is part of a new line of research on the acquisition of different discourse-related positions in the left periphery of the clause identified by cartographic studies (Rizzi and Bocci, 2017). Specifically, the research aimed at studying the acquisition of Topic positions hosting a left dislocated direct object, which in Italian/Romance yields a so called Clitic Left Dislocation/ClLD as in *Il cane, il gatto lo lava*/the dog, the cat washes it-Cl. In ClLD the sentence following the left dislocated direct object, which is a discourse topic given in previous discourse, predicates a property of the preposed noun phrase and (obligatorily) contains a clitic pronoun referring to it (*lo* in the example above). The second domain is a classical research topic in language acquisition, which has recently received renewed attention in the theoretical debate (see, a.o. Manetti, 2013; Snyder and Hyams, 2015; less recently Crain, 1991), i.e. the acquisition of passive, in the aim of determining which types of passive children may prefer or access first, if there is any preference or earlier access at all.

In what follows, I will briefly outline the essential relevant features of the experimental design and illustrate and discuss aspects of the results that are relevant for the present discussion. For a thorough description (of both method and materials) and overall discussion of the articulated results the reader is referred to Belletti and Manetti (2017). Overall, the results to be reviewed here provide new evidence from empirical domains that have not been previously discussed in this connection, that children's linguistic behavior does not simply mirror adult production and does not simply reflect what children hear most. In this sense then, children are capable to express an intriguing grammatical creativity that does not conform to their input (pace Tomasello, 2003, and subsequent related literature). Such creativity in turn is not unconstrained, but, as will be illustrated here, follows the principled organization of the UG-constrained internal grammatical system, thus also indicating clear continuity in the process of linguistic development.

## Outline of the design and of main relevant results

In Belletti and Manetti's (2017) design (their Experiment 1) young children (39, age range 4;1–5;11) had to answer a question concerning the object of a transitive action. As mentioned in the introduction, the aim of the elicitation experiment was to check whether young children at the ages investigated can access left peripheral topic positions; a related aim was to also determine whether they can access passive structures and, in case they do, which type of passive they prefer among the different ones available in Italian, if there is any preference at all. The question came at the end of a short introductory story, which was accompanied by illustrating images. For instance, given a story ending with a picture showing “a giraffe licking a cow and a rabbit touching a penguin,” in a Two-topic condition (i.e., a contrastive topic situation, Benincà and Poletto, 2004; Bocci, 2013), the question in (1) was asked to the children:

(1)  Che cosa succede ai miei amici, il pinguino e la mucca?
       What is happening to my friends, the penguin and the cow?

Italian speaking children (both age groups) often answered to this type of question with a ClLD structure (25% of their answers). Use of a ClLD structure in this discourse condition is perfectly adequate and appropriate. However, children realized the ClLD in a peculiar way: Children's preposed direct object was typically introduced by preposition *a*, as illustrated in (2):

(2)  *Il coniglio a i' pinguino lo tocca*
      The rabbit to the penguin him.Cl touches
     “The rabbit touches the penguin”        (Adele 4;9)

I will refer to this type of preposed direct object topic as an *a*-Topic. Crucially, all children were monolingual speakers of a central (Tuscan) variety of Italian. In this variety, which corresponds to the standard one, direct objects are not introduced by preposition *a*.

Another type of answer produced by children in some cases (11% of their answers) is a passive sentence. This type of answer is also perfectly adequate and appropriate in the discourse condition created by the experiment; in fact, this is the type of answer most widely adopted by the 24 adult controls (68% of their answers), in exactly the same elicitation experiment. The passive utilized by children, however, is different from the one most typically produced by adults. Children exclusively resorted to a type of passive that is rarely present in adult Italian, a reflexive-causative passive illustrated in (3) (*si*-causative passive henceforth):

(3)  *La mucca si fa leccare dalla giraffa*
      The cow SI-makes lick by the giraffe
     “The cow makes the giraffe lick it”      (Olmo, 4;1)

And indeed also in the experimental setting of the experiment, the most widely adopted type of passive in adults' answers was not a *si*-causative passive (19% of the produced passives) but rather a periphrastic passive using *essere*/be or (mostly) *venire*/come as the passive auxiliary (49% of the produced passives), as in (4) (copular/*venire* passive, henceforth):

(4)  *La mucca è/viene leccata dalla giraffa*
      the cow is/comes leaked by the giraffe
     “The cow is being licked by the giraffe”

In the following sections I discuss and motivate in some detail the relevance of these results for the issue raised in the introduction concerning children's grammatical creativity.

## The case of *a*-topics in children's CLLDs

In standard Italian direct objects are not introduced by a preposition, no matter what their nature is (e.g., specific or indefinite). Standard Italian is not a so-called Differential Object Marking/DOM language. Only in few cases, and marginally so for many speakers, can direct objects be realized as *a*-Topics: when they are the object experiencer of psych-verbs of the *worry* class. See the following contrast in (5), from Belletti and Rizzi (1988), quoting Benincà's observation (Benincà, 1986).

(5)     a (?)A Gianni, questi argomenti non l'hanno convinto
               to Gianni, these arguments him-CL have not convinced
            “  These arguments have not convinced Gianni”
          b ^*^A Gianni, la gente non lo conosce
               to Gianni, people him-CL do not know
             “People do not know Gianni”

The contrast between (5)a, marginally acceptable for some speakers, and (5)b completely excluded by all Italian speakers illustrates the fact that only an object experiencer can be (marginally) realized as an *a*-Topic. No contrast is present in (6) in which the object fills the object position and is not preposed; *a-*marking is excluded in both cases:

(6)    a ^*^Questi argomenti non hanno convinto a Gianni
              these arguments have not convinced to Gianni
           b ^*^La gente non conosce a Gianni
              people do not know to Gianni

The examples in (7) illustrate the other context in which *a*-Topics are possible in standard Italian: when the topic is a (mainly 1st or 2nd person) pronoun, possibly also allowing, in these cases, direct objects that are not experiencers (see also Renzi, 1988; Berretta, 1989 for relevant discussion):

(7)   a A te, non ti conosco
         to you I do not you-Cl know
         “I do not know you”
       b A me, nessuno mi ha chiamato
          to me nobody me-Cl has called
         “Nobody called me”
       c ?A lui, lo rispettano
          to him/him they him-CL respect
         “They respect him”

These are the two main distributional properties of *a-*Topics in standard Italian. A detailed discussion and description of the constrained distribution of *a*-Topics in Italian is provided in Belletti (2017a) where the hypothesis is put forth that *a*-Topics may be the realization of a property of the left periphery whereby the preposed object is interpreted as affected by the event described by the verb and the speaker feels particularly involved and adopts an empathic point of view towards it^1^. Thus, by expressing the preposed object in the form of an *a*-Topic, children have overextended the constrained and limited option of adult standard Italian in at least two ways:

- All of their *a-*Topics were lexical noun phrases (i.e., they were not-−1st or 2nd person—pronouns)
- All of their *a-*Topics were objects of agentive verbs (i.e. they were not object experiencers of psych-verbs)

In children's experimental results from Belletti and Manetti (2017, Experiment 1), direct object topics have been realized as *a-*Topics in the vast majority of cases. Specifically, when the preverbal subject of the following clause was an overt lexical noun phrase, the topic was realized as an *a*-Topic in 88% of the cases^2^; it was realized as a simple direct object topic instead (with no preposition) in the remaining 12% of the cases. This is illustrated in (8). Recall that in standard Italian the latter option in which the preposed object topic is not introduced by preposition *a* (e.g., *Il coniglio il pinguino lo tocca*/the rabbit the penguin it—Cl touches) would be the only possible option with agentive verbs, as were all of the verbs used in the experiment.

(8) *a*-Topics in presence of lexical pre-verbal subjects


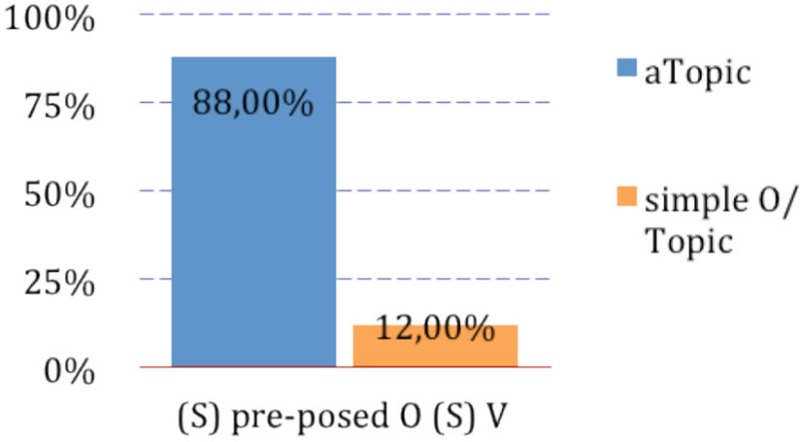


The realization of the preposed object topic as an *a-*Topic clearly correlates with the nature and position of the subject. This is shown by the fact that in some cases children used either a null subject or a post-verbal subject. Both options are grammatical in a null subject language like Italian, although the discourse conditions favored the overt and preverbal realization of the lexical subject, indeed the most widely adopted option by children. However, in those cases in which children opted for the null or post-verbal realization of the subject in the clause following the left dislocated object topic, the latter has been realized either as an *a-*Topic or as a simple Topic, with no *a* in an almost identical proportion. (9) Illustrates the distribution of *a-*Topics and simple Topics according to the nature and position of the subject:

(9)  Comparing *a*-Topics and simple/O-Topics according to the nature (lexical or null) and position (pre-verbal or post-verbal) of the subject.
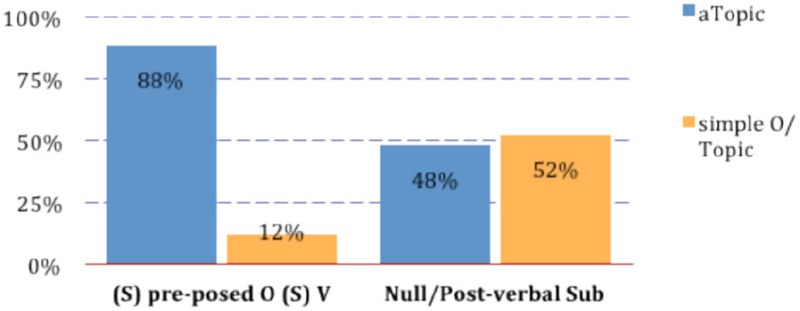

Why should the nature and position of the subject influence the realization of the Topic as an *a-*Topic? A principled reason can be assumed to be the origin of this influence. As discussed in detail in Belletti and Manetti (2017) ClLD structures of the type in (2) under investigation display an object A'-dependency across an intervening lexical pre-verbal subject, in which both the preposed object and the subject are lexically restricted. According to the system developed in Friedmann et al. (2009), the notorious difficulty that children encounters with object A'-dependencies involving this intervention configuration—as in e.g. headed Object relative clauses with a pre-verbal lexical subject in the relative clause—may be accounted for in terms of the grammatical principle Relativized Minimality-RM expressed in featural terms, fRM (Rizzi, 1990, 2004; Starke, 2001; Grillo, 2008 for the proposal that the principle may also account for aspects of the agrammatic behavior in aphasia, on which see also Sheppard et al., 2015). According to the to the featural RM principle, in a configuration such as:

(10) 
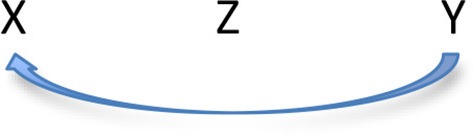


in which a dependency relation between the target position X and the origin position Y has to be established across an intervening Z^3^, such dependency cannot be established if X (target) and Z (intervener) share relevant features. In movement created dependencies, the relevant features are those triggering the displacement operation and attracting the relevant constituent in the target position. For instance, according to Friedmann et al. (2009), in headed object relatives the features attracting the relative head into the complementizer domain are a [+R] feature and a [+NP] feature. If a lexically restricted subject is present in the relative clause it also carries the [+NP] feature. Thus, by expressing the feature relations in set theoretic terms, the lexically restricted relative head (X), *il cane* of example (11), and the intervening lexically restricted subject (Z), *il gatto* in the same example (11), are in a relation of inclusion, with the feature [+NP] of the intervening lexical subject properly included within the feature set of the target.

(11) *Il cane      che    il gatto   morde* ___
        the dog        that   the cat    bites
        +R+NP              +NP
        X                          Z                     Y

Indeed, if either the head of the object relative is not lexically restricted, as in the case Free object Relatives, or the subject of the relative clause is not lexically restricted as in the case in which it is a pronoun, object relatives are well understood by children, at the same level as subject relatives. This is the core experimental finding of Friedmann et al. (2009), which the system captures through the proposal that there is development in the proper computation of the inclusion relation of the features which are relevant for the fRM principle. Further work has shown that the intersection relation of features relevant for the principle can be properly computed by young children (Belletti et al., 2012). Thus, for instance, illustrating once again with Italian, whereas a headed object relative like (11) is poorly comprehended by young children until a late age (still at 8-9 y.o. see Adani et al., 2010; Adani, 2011; Contemori and Belletti, 2014), object relatives in which the relative head and the subject of the relative clause mismatch in number are properly understood by children in a significantly higher proportion (Adani et al., 2010 for the relevant results on the mismatch configuration). This situation instantiates the intersection configuration, as (12) illustrates:

(12) *Il cane      che    i cavalli     rincorrono* ___
          the dog      that   the horses   run after
         +R+NP ±sing       +NP + pl
         X                            Z                            Y

Thus, according to the system in Belletti et al. (2012), grounded on Friedmann et al. (2009), given the four set theoretic relations, disjunction in relevant features is well-processed by both children and adults, identity is excluded by both (the core cases of classical RM, Rizzi, 1990); intersection is also well processed; in contrast, there is development in the proper computation of the inclusion relation of those features that the principle takes into account. The hypothesis is that such features are those that trigger syntactic movement, such as A'-movement into the left periphery of the clause. Thus, given this background, going back to the ClLD structure under investigation here, the proposal can be made that resort to *a*-marking of the object Topic in the ClLD structure containing a preverbal lexical subject is able to create an intersection relation between the feature composition of the target (X)—the left dislocated direct object—and of the intervener (Z)—the lexical subject. Under the assumption that *a*-Topics are associated with an affected interpretation of the topic a feature dubbed [+a] (affected, Belletti, 2017a) can be associated to an affected topic and a complementary feature [+u] to an unaffected one. The following intersection of relevant features illustrated in (13) is thus created, complying with the fRM principle (Belletti and Manetti, 2017, for all further relevant details):

(13)   *Il coniglio       al pinguino      lo           tocca*
            The rabbit        to the penguin   him.Cl   touches
         +Top +NP +u    +Top+NP +a

In conclusion, it seems that a number of reasons may (have) contribute(d) to make *a*-Topics favored by young children in the results reviewed. Among them the following two play a crucial role:

- The fact that the preposed object, with which children establish an empathic relation is compatible with the (psychologically) affected interpretation associated with left peripheral *a*-Topics
- The fact that, in presence of a lexically expressed overt subject, resort to *a*-Topic effectively modulates the intervention problem posed by the syntactic configuration.

In contrast, frequency in the input of these structures appears to be an un-influential factor. As discussed, such structures do not really “exist” in standard Italian in the form widely adopted by the children. As the comparison in (9) strongly suggests, the crucial factor determining children's overextension in the use of *a*-Topics is the internal grammatical pressure of coping with a complex structure such as the one manifesting the hard intervention configuration^4^

## The case of *si*-causative passive in children's passives

As mentioned in the introduction, sometimes children's answer to the question on the object of Belletti and Manetti's (2017) Experiment 1 that is reviewed here was a passive sentence (11%). Passive is a further appropriate type of answer given the experimental conditions, which corresponds in fact to the adults' widely adopted option (68%). See Belletti and Manetti (2017) for proposals on the possible reasons accounting for the difference between children and adults in the selection of the preferred answer to the elicitation question, which ultimately indicates that passive is not yet productively mastered at the ages under investigation as children's preferred answer in the same conditions were the ClLD structures discussed in section The Case of a-topics in Children's Cllds^5^. Here, I would rather like to focus on the comparison between children and adults as for the types of passive utilized by the two groups in light of the issue of children's unexpected linguistic behavior under discussion here.

As is clearly illustrated in (14), children's and adults types of passives differ considerably: children exclusively selected the *si*-causitive passive (all their 11% of passive answers were *si-*causative passives), whereas the most frequently utilized passives by adults were the periphrastic ones, copular or *venire* passive (49% out of their 68% of passive answers)^6^.

(14) Different types of passives produced by adults and children


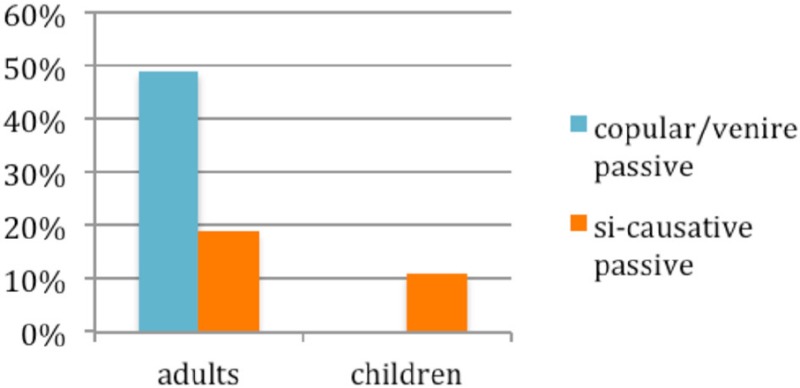


The somewhat privileged status of *si*-causative passive in Italian speaking young children had also been found in previous experiments, using different techniques and eliciting different structures (e.g., *syntactic passive priming*, Manetti and Belletti, 2015; elicitation of object relatives through preference or picture description tasks, Contemori and Belletti, 2014). Let us now concentrate here on the significance of the sharp contrast revealed by figure (14) for the issue under investigation in this article.

We note first of all that the contrast in (14) cannot be due to children's sensitivity to the frequency of *si*-causative passive in their Italian input, since, as argued in Belletti (2017c), this type of passive is in fact rather rare compared to copular and *venire* passives in Italian^7^. Moreover, the computation involved in *si*-causative passive looks intuitively rather complex in that, beside including aspects of the computation also at play in copular and *venire* passive such as the *smuggling* operation moving a chunk of the verb phrase (Collins, 2005), it also involves one extra verb, the causative verb *fare* and the reflexive clitic *si* with the binding relation that its presence induces. However, far from being factors increasing the complexity of the computation, these grammatical properties are probably among those that make the *si*-causative passive more readily accessible to young children: on the one hand, the *smuggling* operation overtly triggered by the causative verb *fare* allows for a derivation in which intervention is properly eliminated (Manetti and Belletti, 2015) and on the other presence of the reflexive may constitute a further facilitating factor (Belletti, 2017b on the possible role of the reflexive, inducing a reflexive passive as a route to other types of passives; Belletti and Manetti, 2017 for further elaboration of these points). Thus, the robust access to *si*-causative passive that children have shown in this experiment, and which confirms previous independent results, indicates once again that children do not always do what they hear most. Furthermore, they also show early mastery of computations which are neither shorter nor simpler in any pre-theoretical sense, but which must count as such for their internal grammar.

## Conclusions

The following conclusions can be drawn from the acquisition results reviewed here.

Grammatical and discourse related factors may sometimes lead children to systematically choose (the production of) structures which are only marginally present in the adult language. Overall, there does not seem to be any penalty for young children to access apparently complex and long(er) expressions *per se*, which can in fact sometimes be favored, as in the two cases reviewed. Both the *a-*Topics and the *si*-causative passives that children produced do involve longer expressions: a simple (object) topics without preposition *a*, is shorter than an *a*-Topic. Similarly, *copular* or *venire* passives, which do not involve the extra causative verb *fare* nor the reflexive clitic *si* with the implied binding relation are shorter and look simpler than *si*-causative passive. In both cases, however, the extra lexical elements may allow children to implement computations, which are in fact more readily accessible to their developing grammatical system.

Children thus end up displaying a grammatical behavior, which differs sharply from that of adults, as it happened in both cases considered here. Children's capacity to overextend given syntactic structures thereby resulting in a grammatical creative behavior is the sign of an internal grammatical pressure, which does not necessarily require a rich input to be put into work^8^. The experimental conditions have succeeded in highlighting the children's grammatical creativity in newly identified contexts.

## Author contributions

The author confirms being the sole contributor of this work and approved it for publication.

### Conflict of interest statement

The author declares that the research was conducted in the absence of any commercial or financial relationships that could be construed as a potential conflict of interest.

## Footnotes

^1^A comparison with Spanish is also provided in Belletti (2017a), mainly based on Leonetti (2004) and references cited therein. A discussion of the comparative issue goes beyond the scope of the present article. It can just be noted in passing here that southern varieties of Italian are DOM languages along similar lines as Spanish. Hence, for speakers of these varieties *a*-Topics would be the reflex of DOM. A totally different situation from the one described in the text, as children were all speakers of the standard non-DOM variety of Italian as they never marked the direct object in its canonical direct object clause internal position with preposition *a*. According to Escandell-Vidal (2009) in Balearic Catalan direct objects are *a*-marked when they are (left peripheral) topics, but never when they fill the canonical direct object position, thus displaying the same syntax as the one of the Italian speaking developing children discussed in the text. This is an interesting convergence with the results from the Italian-speaking children, which also further illustrates the UG-constrained continuity of language development mentioned in the introduction. This comparative aspect of the significance of the results discussed here is further developed in current work.

^2^Note that the lexical subject may either precede (as in example 2) or follow the *a*-Topic (e.g., compared to 2: *al pinguino il coniglio lo tocca*/to the penguin the rabbit him-Cl touches); in the former case it also fills a left peripheral topic position, multiple topics being possible in Italian (Rizzi, 1997; Frascarelli and Hinterhölzl, 2007 a.o.); in the latter case it may either fill a Topic position lower than the *a*-Topic or be in the preverbal subject position within the clause (i.e., Spec/TP). The two possible orders are abbreviated as (S) preposed O, (S) in the Figure in (8).

^3^Where the intervention situation relevant to the principle is not linear but hierarchical, such that X c-commands Z, Z c-commands Y, and does not c-command X. The hierarchical nature of the relation is motivated on a vast amount of evidence in the theoretical linguistic literature. See Rizzi (2013) for recent discussion.

^4^Next to (13) the following order is also possibly realized in which the object *a-*Topic precedes the lexical subject (see footnote 2). The latter can thus fill either a peripheral topic position below the preposed object or the clause internal subject position. Nothing changes as for the intersection relation of relevant features:

*   Al re             il bambino*         __ *lo sta pettinando__*
    +Top +NP +a (+Top) +NP +u
     (to the king    the boy     him-CL is combing

As shown in Costa et al. (2014) for PP relatives in European Portuguese and Hebrew, the categorial distinction DP vs. PP connot be considered the relevant distinctive factor.

^5^Adults did not produce ClLDs in their answers (1%) as the passive answer took clear priority for them. This recalls the same adults' behavior found in previous work (Contemori and Belletti, 2014) whereby the production of relatives in the passive (i.e., Passive Object Reatives/PORs) in place of active object relatives was the overwhelmingly adopted answer by Italian speaking adults.

^6^More the latter than the former, as is natural with the actional verbs of the experiments all in a simple tense (present), the two fundamental conditions regulating *venire* passive.

^7^And indeed passive adults' answers to the elicitation question confirm that *si-*causative passive is not the passive that adults resort to most, as indicated in (14). Possibly their *si-*causative passive answers involved a causative/agentive type interpretation as it is generally the case for *si*-causative passives in adult standard Italian. It is hard to say whether the same interpretation is also necessarily at work in children's answers or whether their *si*-causative passive is just a form of passive with no necessary causative interpretation involved (as it is the mainly the case in e.g., standard French). See also the references quoted in the text on these points.

^8^Nor does it appear to require special external conditions, such as e.g., multilingualism, contacts etc. The reported results all came from monolingual children speaking the same central Tuscan variety of Italian.
